# Report of Two Pulmonary Sarcomatoïd Carcinoma Cases With Highlights on the Computed Tomography Features

**DOI:** 10.7759/cureus.16935

**Published:** 2021-08-06

**Authors:** Narjisse Aichouni, Christine Kora, Afaf Thouil, Hatim Kouismi, Rachid Marouf, Imane Kamaoui, Siham Nasri, Imane Skiker

**Affiliations:** 1 Radiology, Mohammed VI University Hospital, University Mohammed First, Faculty of Medicine and Pharmacy, Oujda, MAR; 2 Pneumology, Mohammed VI University Hospital, University Mohammed First, Faculty of Medicine and Pharmacy, Oujda, MAR; 3 Thoracic Surgery, Mohammed VI University Hospital, University Mohammed First, Faculty of Medicine and Pharmacy, Oujda, MAR

**Keywords:** sarcomatoïd carcinomas, lung, computed tomography scan, diagnostic, case report

## Abstract

Pulmonary sarcomatoïd carcinomas are a heterogeneous group of poorly differentiated non-small cell tumors with a sarcomatous component. On imaging, they appear as peripheral or central masses, sometimes excavated. We report two cases of pulmonary sarcomatoïd lung carcinoma. The first case involves a 73-year-old active smoker who presented with dyspnea. A computed tomography (CT) scan showed a large locally advanced left lower lobar tumor process. A CT-guided biopsy was performed and the histopathological examination concluded a pulmonary sarcomatoïd carcinoma. The second case involves a 52-year-old chronic smoker who presented with hemoptysis. CT pulmonary angiography showed an excavated right upper lobar tumor. Histologic work-up of the right upper lobectomy piece objectified a pulmonary sarcomatoïd carcinoma. Pulmonary sarcomatoïd carcinoma has a nonspecific appearance on imaging and should be a part of imaging differential diagnoses in front of a large, lobulated, highly invasive lung tumor with or without excavation.

## Introduction

Sarcomatoïd carcinoma is an uncommon and poorly understood tumor that can affect any organ, but the lung is the most common site. At the pulmonary level, it belongs to the group of non-small cell bronchial carcinomas. The latest World Health Organization (WHO) classification (2004) distinguishes five histological subtypes in this group that represent a continuum of mesenchymal and epithelial differentiation: carcinosarcoma, giant cell carcinoma, spindle cell carcinoma, pulmonary blastoma, and pleomorphic carcinoma [1]. The diagnosis is pathological and requires good sampling of the tumor. These tumors are highly aggressive with a significant metastatic potential and a high risk of recurrence. Regardless of the organ involved, this entity is known to have a very poor prognosis and to be resistant to chemotherapy. Due to its rarity and pleomorphism, its imaging aspect is rarely reported in the literature. We report two cases of pulmonary sarcomatoïd carcinoma (PSC) and discuss the radiological characteristics of these tumors.

## Case presentation

Case 1

**Figure 1:**
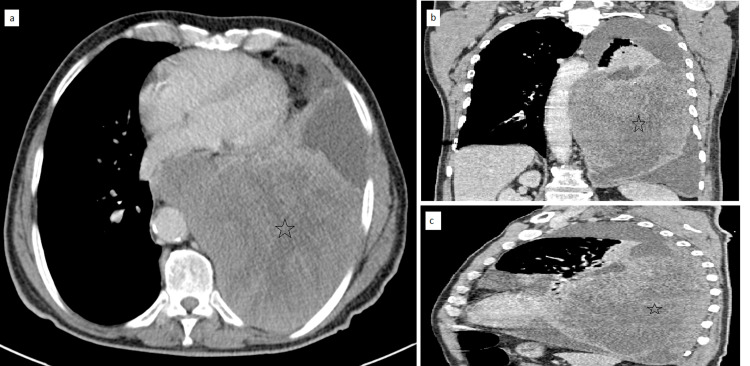
Axial (a), coronal (b), and sagittal computed tomography (c) scans showed a voluminous left lower lobe tumor process (black star) with lobulated margins, heterogeneous, and enhanced after contrast, measuring approximately 135 x 116 x 25 mm.

 A 73-year-old active smoker with no medical history was admitted to our radiology department for investigation of chest pain and dyspnea stage III with deterioration of the general condition (weight loss of 10 kg over the preceding two months). Physical examination objected digital hippocratism and left fluid effusion syndrome. A chest X-ray performed showed left lower lobe opacity with ipsilateral fluid effusion. Computed tomography (CT) imaging (Figure 1) revealed that there was a voluminous left lower lobe lobulated mass, poorly limited, heterogeneous, containing calcifications, and enhanced after contrast. It was locally advanced with intimate contact with the large vessels of the mediastinum, tracheobronchial tree, esophagus, and chest wall. Mediastinal lymphadenopathy and abundant pleural fluid effusion were also associated. After an inconclusive bronchial fibroscopy, a CT-guided biopsy was performed and it revealed a PSC, and the clinical stage was cT4 cN2 cMO. The patient is currently on chemotherapy.

Case 2

**Figure 2:**
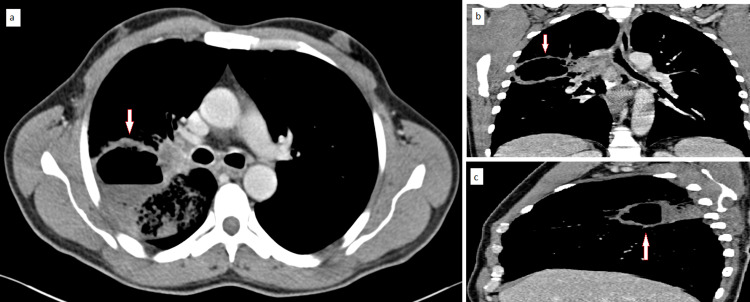
Axial (a), coronal (b), and sagittal (c) computed tomographic scans showing a large cavitating heterogeneous mass of the right upper lobe (arrow) with lobulated margins in the lower lobe of the right lung measuring approximately 8.7 × 8.2 × 7.4 cm.

A 52-year-old patient, a heavy smoker with 36 pack-years of tobacco consumption, was admitted to the emergency department for moderate hemoptysis without any notion of fever, dyspnea, or weight loss. A thoracic angioscan was performed showing a large, irregularly contoured, thick-walled, excavated tumor mass in the right upper lobe that was heterogeneously enhanced after injection (Figure 2).

**Figure 3:**
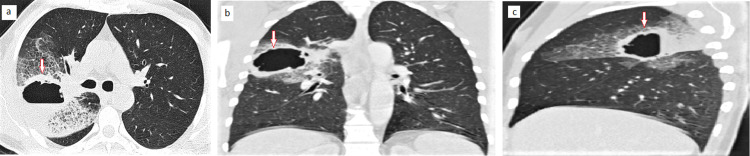
Chest computed tomographic scan axial (a), coronal (b), and sagittal (c) reformatted images, showing a large cavitating mass (arrow) associated with peritumoral reticular infiltrates.

This mass was surrounded by a ground-glass area with reticular infiltrates (Figure 3).

The patient underwent a right upper lobectomy with ipsilateral hilar and mediastinal lymph node dissection. Histological examination of the surgical specimen revealed a spindle cell and large pleomorphic cell proliferation involving the bronchiolar and alveolar structures, which showed an atypical lining with the presence of eosinophilic necrosis. This appearance was suggestive of sarcomatoïd carcinoma. The clinical stage was cT4 cN2 cMO and the patient is currently undergoing concomitant radiochemotherapy.

## Discussion

PSC is a non-small cell lung cancer that has poorly differentiated cells as well as sarcoma or sarcomatoid components (giant cells and/or spindle). PSC is estimated to account for 0.3-1.3% of all lung cancer cases [2]. These tumors have been reported to be smoking-associated. There is a male-to-female ratio of approximately 4:1 [3]. Based on the classification of the WHO, PSC can be divided into five histologic subtypes: carcinosarcoma, giant cell carcinoma, spindle cell carcinoma, pulmonary blastoma, and pleomorphic carcinoma [4]. The clinical signs are not specific and depend on the location of the tumor. Two forms are classically described. A central endobronchial form that is responsible for cough, hemoptysis, and dyspnea as reported in case 2. The second form is peripheral, with a bad prognosis, responsible for pain following the invasion of the chest wall as reported in case 1. An incidental radiological PSC has been reported in 6.7% of cases [5].

The radiological appearance of PSC is not very specific but has some characteristics. They can appear as a central mass like a polypoid endobronchial tumor as described with the pleomorphic carcinoma and carcinosarcoma subtypes. They can also appear as a voluminous (over 10 cm) peripheral mass with rounded, well-defined margins, heterogeneous with necrotic and/or hemorrhagic areas as reported in our patient (case 1). They may be excavated as reported in our patient (case 2) or not and can invade the pleura and thoracic wall at an early stage [6].

Lung, brain, adrenal glands, pleura, and bone metastases are common. Skin metastases have also been recorded [7]. For localized tumors, surgery remains the sole treatment and appears to provide adequate local control. The targeted therapy may be an option for the epidermal growth factor receptor mutation patients. Given the tendency to early metastasis and the high relapse rate, a more aggressive treatment strategy may be needed [8]. Its prognosis is poor (the median survival is about 10 months and the estimated five-year survival rate is 15% [9]), owing to early metastases occurrence, its aggressive behavior, and a high level of chemoresistance to platinum-based standard regimens. The same is true for lung adenocarcinoma with sarcomatoïd transformation due to aberrant activation of the hepatocyte growth factor receptor gene (MET) and expression of programmed death-ligand 1 (PD-L1). MET and PD-L1 inhibitors offer the possibility to manage those patients despite their drug resistance, whose potential also needs to be further studied [9]. PSCs tend to grow rapidly and invade the adjacent structures in the early stage. Several favorable prognostic factors have been reported, including complete surgical resection, no lymph node metastases, lower pathologic stages, and tumor necrosis. On the other hand, pleural involvement was reported as an adverse prognostic factor [8].

## Conclusions

PSCs of the lung refer to a heterogeneous group of rare and poorly differentiated types of non-small cell lung carcinomas. In the presence of a large, lobulated, highly invasive lung tumor with or without excavation, the possibility of a sarcomatous component should be considered. The final diagnosis is made histologically by biopsy or surgical resection of the tumor. Surgical resection remains the cornerstone of the treatment and may offer the possibility of long-time survival in early-stage patients.
